# The COVID-19 pandemic: the public health reality

**DOI:** 10.1017/S0950268820002216

**Published:** 2020-09-22

**Authors:** S. Cheema, M. Ameduri, A. Abraham, S. Doraiswamy, R. Mamtani

**Affiliations:** 1 Institute for Population Health, Weill Cornell Medicine-Qatar, Doha, Qatar; 2 Premedical Education, Weill Cornell Medicine-Qatar, Doha, Qatar

**Keywords:** Coronavirus, COVID-19, pandemics

## Abstract

The coronavirus disease (COVID-19), while mild in most cases, has nevertheless caused significant mortality. The measures adopted in most countries to contain it have led to colossal social and economic disruptions, which will impact the medium- and long-term health outcomes for many communities. In this paper, we deliberate on the reality and facts surrounding the disease. For comparison, we present data from past pandemics, some of which claimed more lives than COVID-19. Mortality data on road traffic crashes and other non-communicable diseases, which cause more deaths each year than COVID-19 has so far, is also provided. The indirect, serious health and social effects are briefly discussed. We also deliberate on how misinformation, confusion stemming from contrasting expert statements, and lack of international coordination may have influenced the public perception of the illness and increased fear and uncertainty. With pandemics and similar problems likely to re-occur, we call for evidence-based decisions, the restoration of responsible journalism and communication built on a solid scientific foundation.

## Introduction

The number of confirmed infections with SARS-CoV-2 has reached 20 million worldwide, and mortality from COVID-19 is estimated to be above 850 000 [1]. All the evidence thus far available quite clearly shows that those at highest risk of a severe illness and death are the elderly, individuals with existing co-morbidities and the immunocompromised. Undeniably, the COVID-19 pandemic has resulted in loss of human life; it has wreaked havoc on healthcare systems worldwide, highlighting inequities in healthcare availability and access; it has resulted in drastic public health measures in most countries of the world. Low- and middle-income nations with weak health systems, dwindling economies, high population density, a high reliance on informal employment, poor technological infrastructure and the double burden of non-communicable and communicable disease are, in particular, more vulnerable to the COVID-19 challenge than high-income nations.

As additional information about the infection and its effects becomes increasingly available, a number of questions which require an explanation arise. While these questions might have been premature a few months ago when very little was known about the epidemiology of the infection, in this commentary we argue that they are now very timely and that it is imperative these questions be addressed. The questions we specifically explore are: How serious is the COVID-19 pandemic? How does it compare with the death burden from other causes? What have been the indirect health and social effects of the COVID-19 pandemic? We also raise questions surrounding misinformation and its negative consequences on health. In exploring these questions and seeking possible answers, we first present data in two parts: (a) epidemiology of COVID-19 and (b) comparison of COVID-19 mortality with mortality from previous pandemics and other causes (for comparison, at the time of writing this paper, the total number of worldwide documented cases and deaths are 27 510 526 and 897 231, respectively) [1]. Subsequently, we summarise the indirect repercussions of the COVID-19 pandemic on non-communicable diseases, economy and lives of people. In the conclusion, we offer a few comments, share thoughts and raise some questions to help open a debate.

## Epidemiology of COVID-19

Based on a review of recent COVID-19 literature, it is clear that the disease is minor in most cases [2, 3]. The estimated infection fatality rate is in the range of 0.66–1.33% [4, 5]. The most recent systematic review and meta-analysis found a pooled infection fatality of COVID-19 to be around 1% among studies with a low risk of bias (Meyerowitz-Katz and Merone, 2020, unpublished). The COVID-19 case fatality rate, in principle an indicator of the virulence of the virus and severity of disease, has been a subject of debate. We now know that this rate may not accurately reflect the true infection fatality rate for a variety of reasons, examples of which include inadequate testing, the high number of mild/asymptomatic cases and failure to include those cases in computing the final rate and the country-specific methods of attributing deaths to COVID-19. A number of recent studies, primarily in the USA and in Spain, which used antibody testing of population samples indicate that the number of undocumented infections is significantly high. These undocumented infections are often not included in computing the published case fatality rates. While the epidemiological implications of these results remain uncertain, they nevertheless strongly suggest that the infection fatality rate is much lower than the currently reported crude case fatality rate of 3.67% [4]. Data are becoming available on the number of deaths per million population in the World Health Organization (WHO) Weekly Epidemiological Reports. As of 6 September 2020, the WHO reported 855 deaths per million in Belgium, 612 in the UK and 564 deaths per million in the USA [6]. This may be a truer reflection of the severity of COVID-19.

We cannot and should not understate the severe disease paradigm in those at higher risk, which includes elderly individuals and those with underlying chronic conditions such as obesity, diabetes, heart disease, cancer, chronic lung conditions and an immunocompromised status. Additionally, clinical presentation characterised by underlying pathological changes such as thromboembolism, cytokine release and inflammatory syndrome resulting in damage to the lungs, cardiovascular system, liver, kidneys, pancreas and nervous system, have been noted and described [7].

## COVID-19 mortality comparison

Here, we present data that pose questions on the magnitude of attention that the COVID-19 pandemic has garnered compared to other public health issues that are in dire need of prevention and response. Table 1 compares the mortality of COVID-19 with past pandemics of the 20th and 21st centuries. The mortality rate ratios (between past pandemics and COVID-19) ranged from 0.2 times (for the lower estimate of the 2009 ‘Swine flu’ pandemic) to over 483 times (the upper estimate of the 1918 ‘Spanish’ flu pandemic) that of COVID-19, after adjusting for population size.

**Table 1.** Mortality in pandemics of the 20th and 21st centuries

Name	Death per 1000 people	2020 million deaths equivalent^a^	Ratio to COVID-19 today^b^
‘Spanish’ flu 1918–1920 [8–11]	9.44–55.56	73.67–433.33	82.1–483.0
Asian flu 1957–1958 [8, 12–14]	0.35–1.38	2.70–10.80	3.0–12.0
Hong Kong 1968–1969 [8, 12, 14, 15]	0.28–1.13	2.20–8.81	2.5–9.8
‘Swine’ flu 2009–2010 [8, 16]	0.02–0.08	0.17–0.65	0.2–0.7
COVID-19 2020 [1, 8]	0.115	0.90	1.0

a Number of deaths adjusted for world's population in 2020.

b Ratio of number of deaths compared to COVID-19 as of 8 September 2020.

While coronavirus infection and death rates continue to escalate in some communities and decline in others, most experts agree that COVID-19 continues to present a significant risk especially to the elderly and those with chronic conditions.

It should be emphasised that the other causes of death during the COVID-19 pandemic cannot be ignored. According to the Institute for Health Metrics and Evaluation (IHME), non-communicable diseases account for over 41 million deaths globally, while communicable and nutritional diseases claim over 10 million lives [17]. Of the latter, 8.1 million deaths were from HIV/AIDS, tuberculosis, enteric infections, measles and other communicable diseases, most of which are preventable or effectively managed [17]. In 2017, there were 219 million cases of malaria (95% confidence interval (CI): 203–262 million) worldwide, causing an estimated 435 000 deaths [18].

Furthermore, we observe that deaths due to some acute and largely preventable causes far exceed COVID-19-related deaths. IHME 2017 data on mortality suggest that deaths due to injuries exceed those of COVID-19, as of 8 September 2020 [17]. Road fatalities, including motor vehicles, cyclists and pedestrians, account for the largest proportion of these, at over 1.2 million. Over 50% of injury-related fatalities and more than 80% of communicable and nutritional disease-related fatalities occur in low-income and low-middle-income countries. Also, the WHO estimated that in 2019, iatrogenic or medical errors caused 2.6 million deaths in the lower- and middle-income countries alone [19]. These figures demonstrate that there are other concurrent problems causing distressingly high fatality rates that should not be overlooked as we continue to battle the COVID-19 pandemic.

While mortality is an important measure to ascertain the seriousness of COVID-19, its indirect serious health, social and financial consequences cannot be ignored.

## Impact on non-communicable diseases

The presented data also suggest that the world today may be facing bigger public health challenges than COVID-19. Is the world's reaction to the pandemic in terms of lockdown and travel restrictions disproportionate? We express our concern on the impact that these prevention measures have had, particularly on the mental health and livelihood of the poor and the most vulnerable populations. More importantly, the current scenario risks compromising the physical, mental and social health of individuals and communities [20]. There are reports that persons with non-communicable diseases are failing to seek timely care due to fear of breaking lockdown rules, the threat of acquiring COVID-19 during visits to healthcare facilities, and the choice made by hospitals to treat emergencies only [21]. The risk of adverse health effects due to postponement of routine and elective care along with the severe mental stress and depression caused by this largely unprecedented situation is of grave concern. Isolation, unemployment and loss of income may further compound the misery of already lonely individuals and families leading to a rise in self-harm and suicidal ideation, gender-based and domestic violence and the risk of substance use [22].

## Social and economic disruptions

The evidence of the dramatic economic impact of the measures undertaken in many countries to fight the spread of the disease is apparent. For example, in the USA, unemployment is at a record high and the economy is tumbling. Nationwide, women, people of colour and the young are affected the most [23]. The loss of income is likely to result in an increase of adverse health outcomes for many of the individuals affected, and the overall economic crisis will negatively impact the ability of entire countries to provide effective healthcare to their citizens.

For individuals in low- and middle-income nations, loss of income, separation from loved ones and social isolation may be legitimately viewed as a bigger threat to long-term survival than the doom and gloom associated with the COVID-19 pandemic. Such a phenomenon has been observed during the economic crises faced by countries prior to the COVID-19 pandemic. The financial crisis in Greece, for instance, is estimated to have caused an additional 242 deaths per month between September 2008 and December 2013, due to cardiovascular disease, suicide and mental health illness disproportionately affecting women and people older than 65 [24]. Job loss during a recession in the USA was associated with significant increases in mortality (hazard ratio: 1.6; 95% CI 1.1–2.3) [25]. In Brazil, a middle-income country, an analysis by Hone *et al*. determined that a 1% rise in the unemployment rate was associated with 0.50 increase per 100 000 each quarter in all-cause mortality and that unemployment resulted in 31 415 additional deaths between 2012 and 2017 [26]. Hence, we believe that the mortality and disease burden during and after the COVID-19 pandemic due to the social and economic consequences of the preventive measures and other factors can be substantially high. In addition to the direct effects on mortality, it is also feared that the economic disruptions could lead to the doubling of malnourished children in Africa in the next 6–9 months [27]. In a recent interview with the Washington Post, Mark Lowcock, United Nations Undersecretary General for Humanitarian Affairs, said, ‘There's a huge COVID-19 impact which is economic, and that is drowning out the disease itself’ [28]. It is hence critical to have an eye on the overall effects of the pandemic both on the short- and long-term.

## Misinformation and sensationalism

It is hard at this stage to reconstruct the sequence of events leading to the haphazard and incoherent response of most countries to the spread of the pandemic. However, we caution against fear-mongering associated with sensational narratives and inappropriate media reporting, which can result in political pressures that global leaders, policymakers, employers and even some healthcare professionals may have been under, along with the initial uncertainties concerning the severity and nature of the disease.

Sensationalism, confusion stemming from contrasting statements from authority figures and the lack of international coordination have influenced the public perception of the illness, increasing fear and uncertainty. As an example, we cite the hydroxychloroquine saga. The sale of this medication in the USA jumped leaps and bounds with just a mention of its potential benefit from the US President [29]. Similarly, the differing recommendations on the use of masks from the WHO and the US Centers for Disease Control have contributed to the public's confusion [30]. In addition, the pervasive and increasing role that social media play in how people obtain and share information increases the risks of misinformation and confusion.

Misinformation can imperil the health of public in other ways. In a recent online US survey, it was observed that US adults are engaging in more frequent cleaning and disinfection of their home to prevent SARS-CoV-2 infection. The study points out that 40% use cleaning agents or disinfectants in an unsafe manner that presents health risks. For example, 19% reported using bleach on food (fruit or vegetables) and 18% reported using cleaning products on their skin [31].

## Conclusions

We should neither downplay nor overstate the pandemic risk. Those at increased risk of severe disease should receive priority and be effectively managed. From a public health perspective, it is our opinion, that the lack of a timely internationally coordinated evidence-based approach, the inadequate preparedness of health systems and the absence of effective global leadership has driven us to the current health, economic and social disruptions.

The lack of control and coordination over who is saying what, how, where and when, can propel misinformation, leading to fragmented decision-making and public confusion. Should there not be an agreed upon deontological code to discourage sensational reporting? Why are there not globally acceptable guidance statements on commonly used measures such as the use of face masks and chloroquine?

The COVID-19 pandemic continues to evolve. Moving forward and with pandemics likely to re-occur, we call for health decisions to be made on the basis of science and public health evidence. Restoration of responsible journalism and communication driven by scientific truth and valid data is of paramount importance. Imparting public health education in school, college and community settings to inform learners about health, disease risks and general aspects of public health challenges such as infectious diseases is vital.

## Data Availability

The dataset(s) supporting the conclusions of this article is (are) included within the paper.
